# Association Between Synovial NTN4 Expression and Pain Scores, and Its Effects on Fibroblasts and Sensory Neurons in End-Stage Knee Osteoarthritis

**DOI:** 10.3390/cells14060395

**Published:** 2025-03-08

**Authors:** Ayumi Tsukada, Yui Uekusa, Etsuro Ohta, Akito Hattori, Manabu Mukai, Dai Iwase, Jun Aikawa, Yoshihisa Ohashi, Gen Inoue, Masashi Takaso, Kentaro Uchida

**Affiliations:** 1 Department of Orthopaedic Surgery, Kitasato University School of Medicine, Sagamihara City 252-0374, Kanagawa, Japan; amidesutarere9010@yahoo.co.jp (A.T.); uekusa18y@gmail.com (Y.U.); m.manabu0829@hotmail.co.jp (M.M.); daiiwase19760601@yahoo.co.jp (D.I.); jun43814@gmail.com (J.A.); 44134413oo@gmail.com (Y.O.); ginoue@kitasato-u.ac.jp (G.I.); mtakaso@kitasato-u.ac.jp (M.T.); 2 Division of Blood Transfusion and Transplantation, Kitasato University School of Health Sciences, Minamiuonuma 949-7241, Nigata, Japan; eohta@kitasato-u.ac.jp (E.O.); hattori.akito@kitasato-u.ac.jp (A.H.); 3 Program in Cellular Immunology, Graduate School of Medical Science, Kitasato, Sagamihara City 252-0375, Kanagawa, Japan; 4 Center for Cell Design, Institute for Regenerative Medicine and Cell Design, Kitasato University School of Allied Health Sciences, Sagamihara City 252-0374, Kanagawa, Japan; 5 Medical Sciences Research Institute, Shonan University, Chigasaki City 253-0083, Kanagawa, Japan

**Keywords:** osteoarthritis, netrin-4, synovium, sensory neuron, fibroblast

## Abstract

Osteoarthritis (OA) is a chronic joint disease marked by synovial inflammation, cartilage degradation, and persistent pain. Although Netrin-4 (NTN4) has been implicated in pain modulation in rheumatoid arthritis (RA), its role in OA pain remains less understood. Previous research has documented that NTN4 promotes axonal growth in rodent-derived neurons; however, its effects on human sensory neurons are yet to be fully explored. NTN4 also plays a multifactorial role in various non-neuronal cells, such as endothelial cells, tumor cells, and stromal cells. Nevertheless, its specific impact on synovial fibroblasts, which are key components of the synovium and have been linked to OA pain, is still unclear. This study examined the correlation between NTN4 expression levels and pain severity in OA, specifically investigating its effects on human iPSC-derived sensory neurons (iPSC-SNs) and synovial fibroblasts from OA patients. Our findings indicate a positive correlation between synovial *NTN4* expression and pain severity. Recombinant human Netrin-4 (rh-NTN4) was also shown to enhance neurite outgrowth in human iPSC-SNs, suggesting a potential role in neuronal sensitization. Additionally, rh-NTN4 stimulated the production of pro-inflammatory cytokines (IL-6, IL-8) and chemokines (CXCL1, CXCL6, CXCL8) in synovium-derived fibroblastic cells, implicating it in synovial inflammation. Collectively, these results suggest that NTN4 may contribute to KOA pathology by promoting synovial inflammation and potentially sensitizing sensory neurons, thereby influencing the mechanisms of underlying pain.

## 1. Introduction

Knee osteoarthritis (KOA) is a chronic degenerative condition characterized by knee pain, commonly affecting middle-aged and elderly individuals. Pain management has long been a cornerstone of KOA treatment according to established guidelines. Indeed, the 2019 guidelines from the Osteoarthritis Research Society International (OARSI) emphasize the significance of addressing pain as a primary pathological factor in KOA [1]. Therefore, effectively addressing peripheral pain sensitivity is crucial for alleviating KOA-related pain and enhancing the quality of life for patients with this condition.

In human studies on osteoarthritis (OA), inflammation in the synovium contributes to pain through various inflammatory mediators, with evidence linking the severity of synovitis in the knee to pain levels [2,3]. The synovium is richly innervated with nociceptive nerve fibers, which respond to harmful mechanical stimuli and pain-inducing substances. These nociceptors are further sensitized by inflammatory mediators released during OA progression, thereby amplifying the perception of pain [4,5,6,7,8]. This dynamic interplay between inflammation and neuronal sensitization underscores the critical role of sensory innervation in OA pain mechanisms.

Sensory innervation in synovium plays a pivotal role in pain perception, with nerve fibers and their associated receptors directly transmitting pain signals to the central nervous system. The sensitization of nociceptors and their heightened response to pro-inflammatory stimuli contribute significantly to the chronic pain experienced in KOA [9]. Recent advancements in neuroscience have highlighted the involvement of the Netrin family in modulating neuronal sensitization and synaptic plasticity, two processes fundamental to pain transmission and persistence [10,11,12,13]. The Netrin family consists of netrin-1, -3, -4, -5, G1, and G2, which are secreted proteins with diverse biological roles in neural development, axonal guidance, and angiogenesis. Secreted Netrins interact with specific receptors such as DCC (deleted in colorectal cancer), Neogenin, and members of the Unc5 homolog family, while the Netrin-G subfamily binds to unique receptor types [14].

Among the Netrins, Netrin-4 (NTN4), also known as β-netrin, is a laminin-like secreted protein involved in the development of various tissues, including the central nervous system, bones, kidneys, and blood vessels. NTN4 has been extensively studied in the context of axonal guidance, angiogenesis, and tumor biology, with its crystal structure elucidated to reveal insights into its molecular interactions [15,16]. A recent study reported a possible association between NTN4 and pain in patients with rheumatoid arthritis, noting that it augments the branching of sensory neurons [17]. However, the correlation between NTN4 and pain in OA patients remains unclear. Additionally, a previous study evaluated the effect of human recombinant NTN4 on rodent-derived neurons, demonstrating enhanced branching of sensory neurons in vitro [17,18]. Although the homology between human and mouse *NTN4* is 87%, it is still uncertain whether human NTN4 has a similar effect on human-derived sensory neurons. Advances in induced pluripotent stem cell (iPSC) technology have opened new avenues for researching human sensory neurons [19,20]. iPSC-derived human sensory neurons have proven useful for evaluating the function of NTN4 on human sensory neurons.

Recent studies have highlighted the crucial role of synovial fibroblasts in OA pain [21,22]. In healthy joints, these fibroblasts are quiescent, and primarily tasked with producing extracellular matrix proteins to maintain tissue structure and synovial fluid composition. However, in OA, the synovium becomes inflamed, and the fibroblasts within are activated. This activation contributes to pain by sensitizing nociceptive nerve endings in the synovium. Synovial fibroblasts can augment the growth of nociceptive axons and neurites, extending into synovial papillary processes that position nociceptors close to the lining fibroblasts, thereby promoting pain perception.

Previous research has demonstrated that fibroblast-secreted NTN4 facilitates axonal and neurite growth, supporting a mechanistic link between NTN4 and pain propagation in rheumatoid arthritis [17]. In contrast, NTN4 has been shown to influence non-neuronal cells [23,24,25]; for example, our prior studies have reported that NTN4 stimulates the expression of pro-inflammatory cytokine mRNA in stromal cells derived from the infrapatellar fat pad (IPFP) of OA patients [23]. This suggests that NTN4 might also activate synovial fibroblasts through autocrine or paracrine actions. Synovitis mechanisms, including the release of pro-inflammatory mediators such as tumor necrosis factor alpha (TNFα), interleukin 1 beta (IL1β), monocyte chemoattractant protein 1 (MCP1), and IL6, are known to sensitize and activate nociceptors, lowering the threshold required for pain activation and leading to pain sensitization [26,27,28]. These mediators are positively correlated with patient-reported pain and are thought to increase the excitability of sensory neurons [29]. Thus, the potential role of NTN4 in promoting synovial inflammation and activating synovial fibroblasts, directly or indirectly, could be significant in understanding OA pain mechanisms. However, the specific impacts of NTN4 on synovial fibroblasts themselves remain to be fully elucidated.

Our study aims to investigate the correlation between *NTN4* expression and pain levels in OA patients, examining the effects of NTN4 on synovial fibroblasts and human iPSC-SNs.

## 2. Materials and Methods

### 2.1. Study Participants

This study adhered to the Declaration of Helsinki guidelines and received approval from the Kitasato University Institutional Review Board (protocol code: B19-259; approved on 27 January 2020). The sample size was calculated based on the correlation coefficient (ρ = 0.377) observed in the initial 10 cases between NTN4 expression and pain score. After determining the effect size, it was estimated that 50 cases would be required with a significance level of 0.05 and a statistical power of 0.8. Patients diagnosed with KOA based on clinical and radiographic evaluations were recruited for this research. Exclusion criteria included the presence of rheumatoid arthritis, autoimmune diseases, inflammatory arthropathies, systemic joint-affecting diseases, or a history of joint replacement surgery. Synovial tissue samples were collected from KOA patients confirmed by radiography who underwent total knee arthroplasty at our institution. During surgery, tissue samples were obtained from the affected knee. Fifty synovial specimens were rapidly frozen in liquid nitrogen at −196 °C and stored at −80 °C for RNA extraction.

### 2.2. Culture of iPSC-Derived Sensory Neurons and Measurement of Neurite Length

Human induced pluripotent stem cell (iPSC)-derived sensory neurons (iPSC-SNs) were sourced from ReproCELL, Inc. (Yokohama, Japan, Catalog No. RCDN001N). Neurons were plated on 10-mm coverslips coated with poly-L-ornithine and fibronectin in 24-well plates. Cultures were maintained at 37 °C in a humidified atmosphere with 5% CO_2_, and the medium was refreshed every 3 to 4 days. Prior to recombinant human Netrin-4 (rh-NTN4; Cat. no. 1254-N4-025, R&D Systems, Minne-apolis, MN, USA) stimulation, RNA was extracted from the cells to assess the expression of endogenous NTN4 and its receptors using quantitative PCR (qPCR). After 24 h, the neurons were treated with rh-NTN4 at concentrations of 50 and 500 ng/mL. Neuronal morphology images were captured after 4 days of treatment using an Olympus CKX53 inverted microscope. Immunofluorescence analysis was performed on day 14. As described by Ohta et al. [30], neurons were fixed and stained for analysis. Cells were incubated with primary antibodies against TUBB3 (T8660, Sigma-Aldrich, St. Louis, MO, USA) at 4 °C overnight. Following PBS rinsing, the cells were incubated at room temperature for 1 h with Alexa Fluor 647-conjugated secondary antibodies (A-21242, Invitrogen, Carlsbad, CA, USA), with nuclei counterstained using Hoechst 33342 (Sigma-Aldrich). Fluorescence images were acquired using a Keyence BZ-X810 microscope, equipped with a 20× objective lens (NA 0.45; Keyence), a 1.0× optical zoom, and capturing five random fields of view from a single cover glass.

The neurite length of iPSC-SNs was measured in vitro on days 4 and 14. Measurements were performed using the ImageJ (version 1.4.3) plug-in NeuronJ, which was specifically downloaded for this purpose. Neurites extending from the cell body of each neuron were traced, and branched neurites were measured as the total length of all neurites originating from a single cell body. Measurements were taken in two wells for each concentration, with 10 neurons analyzed per well. This process was repeated three times to ensure reproducibility.

### 2.3. Isolation and Culture of Synovium-Derived Fibroblasts

Eight synovial tissue samples were finely minced into small fragments and enzymatically digested using 0.2% collagenase type I (Sigma-Aldrich) in α-minimal essential medium (α-MEM; Nacalai Tesque Inc., Kyoto, Japan) at 37 °C for 2 h with gentle agitation to facilitate cell dissociation. The resulting suspension was filtered through a 100-µm cell strainer (pluriSelect, Leipzig, Germany) to remove undigested debris. The filtrate was centrifuged at 300× *g* for 5 min, and the resulting cell pellet was resuspended in phosphate-buffered saline (PBS). To deplete non-fibroblast populations, the cells were incubated at 4 °C for 30 min with biotin-labeled anti-CD45 (Cat. No. 304004) and CD31 (Cat. No. 536604) antibodies (BioLegend, San Diego, CA, USA), which target hematopoietic lineage cells and endothelial cells, respectively. After antibody incubation, unbound antibodies were removed by washing the cells twice with PBS. Magnetic separation was performed using streptavidin-conjugated magnetic particles (BD Biosciences, Franklin Lakes, NJ, USA) in combination with the IMag magnetic separation system (BD Biosciences). For the separation process, the cell suspension was placed on a magnetic board at room temperature for 8 min to allow binding of antibody-labeled cells to the magnetic particles. The negative fraction, enriched in fibroblasts, was collected. To enhance fibroblast purity, the collected negative fraction was subjected to a second magnetic separation under the same conditions. This sequential separation ensured the effective depletion of CD45-positive and CD31-positive cells, resulting in a fibroblast-enriched population. The isolated fibroblasts were plated in 75 cm² tissue culture flasks (Thermo Fisher Scientific, Waltham, MA, USA) and maintained at 37 °C in a humidified incubator with 5% CO_2_. Cells were cultured in α-MEM supplemented with 10% fetal bovine serum (FBS; Gibco, Thermo Fisher Scientific) and 1% penicillin-streptomycin (Nacalai Tesque). The culture medium was replaced every 3 to 4 days to remove non-adherent cells and support fibroblast proliferation. Prior to rh-NTN4 stimulation, RNA was extracted from the fibroblasts to assess the expression of endogenous NTN4 and its receptors using qPCR. The results were then compared to those obtained from iPSC-SNs. Once cells reached approximately 80% subconfluency, they were detached using 0.25% trypsin/EDTA (Nacalai Tesque) and reseeded into 6-well plates at a density of 2 × 10⁵ cells per well for experimental treatments. After a 3-day incubation period to allow cells to adhere and stabilize, fibroblasts were treated with rh-NTN4 or vehicle control (α-MEM supplemented with 10% FBS). rh-NTN4 was administered at final concentrations of 50 ng/mL or 500 ng/mL. The treatments were performed for 3, 6, or 24 h to evaluate both short-term and long-term effects on fibroblast gene and protein expression. At the conclusion of each treatment period, cells were lysed directly in TRIzol reagent (Invitrogen, Carlsbad, CA, USA) to extract total RNA for quantitative PCR (qPCR) analysis. For protein analysis, cell supernatants were collected after 24 h of treatment and stored at −80 °C for subsequent quantification using enzyme-linked immunosorbent assay (ELISA). In addition, flow cytometry was performed after 24 h of treatment to evaluate VCAM-1 expression (*n* = 8). All experiments were performed with biological replicates, and each experiment was conducted in duplicate to ensure reproducibility.

### 2.4. qPCR

Total RNA was extracted from synovial tissue using MaXtract high-density tubes (Qiagen, Valencia, CA, USA) in combination with the phenol/chloroform method, ensuring high RNA yield and purity. For cultured cell samples, RNA was extracted using the Direct-zol MicroPrep kit (Zymo Research, Orange, CA, USA), following the manufacturer’s protocol, which includes an in-column DNase I treatment step to remove genomic DNA contamination. RNA purity and concentration were assessed spectrophotometrically (Denovix, Tokyo, Japan), ensuring an A260/A280 ratio greater than 1.8 for all samples to confirm RNA integrity. Complementary DNA (cDNA) was synthesized from 0.5 µg of total RNA using Superscript III Reverse Transcriptase (Invitrogen, Carlsbad, CA, USA). Oligo(dT) primers and dNTPs were purchased from Takara Bio (Shiga, Japan). The reaction was carried out in a thermal cycler under the following conditions: primer annealing at 65 °C for 5 min, reverse transcription at 50 °C for 50 min, and enzyme inactivation at 70 °C for 15 min. Primers for the target genes were designed using Primer-BLAST (NCBI) to ensure specificity and efficiency. All primers were synthesized by Hokkaido System Science (Hokkaido, Japan). Primer sequences are listed in Table 1.

Quantitative PCR (qPCR) was performed in a 25 µL reaction volume containing 2 µL of cDNA, 12.5 µL of TB Green Premix Ex Taq II (Tli RNaseH Plus, Takara Bio, Shiga, Japan), 2 µL of each primer, and 8.5 µL of nuclease-free water. Reactions were run in triplicate using the CFX-96 Real-Time PCR Detection System (Bio-Rad, Hercules, CA, USA) under the following cycling conditions: initial denaturation at 95 °C for 10 min, followed by 40 cycles of denaturation at 95 °C for 15 s, and annealing/extension at 60 °C for 1 min. Gene expression levels were normalized to GAPDH as an internal control, and relative expression levels were calculated using the delta-delta CT method. Amplification specificity was confirmed by melt curve analysis.

### 2.5. ELISA

rh-NTN4 was administered to fibroblast cultures at final concentrations of 50 ng/mL or 500 ng/mL. The treatments were performed for 24 h to evaluate protein concentrations in the supernatants. MMP-1 was measured using an ELISA kit (R&D Systems, Minneapolis, MN, USA, Catalog No. DY901B). MMP-3 was also measured using an ELISA kit (R&D Systems, Minneapolis, MN, USA, Catalog No. DY513-05). Additionally, MMP-13 levels were determined using an ELISA kit (R&D Systems, Minneapolis, MN, USA, Catalog No. DY511). Interleukin-6 (IL-6) was measured using an ELISA kit (BioLegend, San Diego, CA, USA, Cat. No. 430515). Interleukin-8 (IL-8) was also measured using another ELISA kit (BioLegend, Cat. No. 431504). Furthermore, CXCL1 was determined using an ELISA kit (ProteinTech, Chicago, IL, USA, Cat. No. KE00133), and CXCL6 was measured with another ELISA kit (ProteinTech, Cat. No. KE00274). All measurements were performed according to the manufacturer’s instructions.

### 2.6. Flow Cytometric Analysis

rh-NTN4 was administered to fibroblast cultures at final concentrations of 50 ng/mL or 500 ng/mL for 24 h. To evaluate VCAM-1 expression, flow cytometric analysis was performed after 24 h of treatment. Cultured fibroblasts were detached using a 0.25% Trypsin/EDTA solution. After removing trypsin, the cells were stained with the following antibodies: APC-conjugated anti-VCAM-1 (BioLegend, Cat. No. 305810), PE-Cy7-conjugated anti-CD90 (BioLegend, Cat. No.328124), and FITC-conjugated anti-CD45 (BioLegend, Cat. No. 304006). The stained cells were analyzed using a FACSVerse flow cytometer (BD Biosciences). Data were processed and analyzed using FlowJo software to calculate the mean fluorescence intensity (MFI) of VCAM-1 expression.

### 2.7. Statistical Analysis

The sample size and statistical power were determined using G*Power 3 software to ensure adequate sensitivity for detecting significant differences and correlations. Data were analyzed using SPSS software (Version 28.0; IBM Corp., Armonk, NY, USA). The Shapiro–Wilk test was used to evaluate data normality within each group. Since the data did not follow a normal distribution (*p* < 0.05), non-parametric tests were applied. The Kruskal–Wallis test, a non-parametric alternative to one-way ANOVA that does not require normality assumptions, was used to evaluate the effects of NTN4 on gene expression in fibroblasts under different treatment time points and concentrations. Post-hoc analysis was performed using the Dunn test to account for multiple comparisons. In addition, Spearman’s rank correlation coefficient was calculated to determine associations between NTN4 expression and VAS scores for active pain. Linear regression analysis was also conducted to examine relationships between NTN4 expression and clinical factors such as age, BMI, K/L grade, and VAS scores. Statistical significance was defined as a *p*-value of less than 0.05.

## 3. Results

### 3.1. NTN4 Expression and KOA Pathology

Table 2 outlines the demographic and clinical characteristics of the 50 study participants, which includes patients across different K/L grades, reflecting various levels of OA severity. Key clinical data recorded for each patient included age, BMI, and pain scores assessed via the Visual Analog Scale (VAS) (Table 2). No significant differences were found in age, BMI, or VAS pain scores between male and female participants (Table 2).

**Figure 1:**
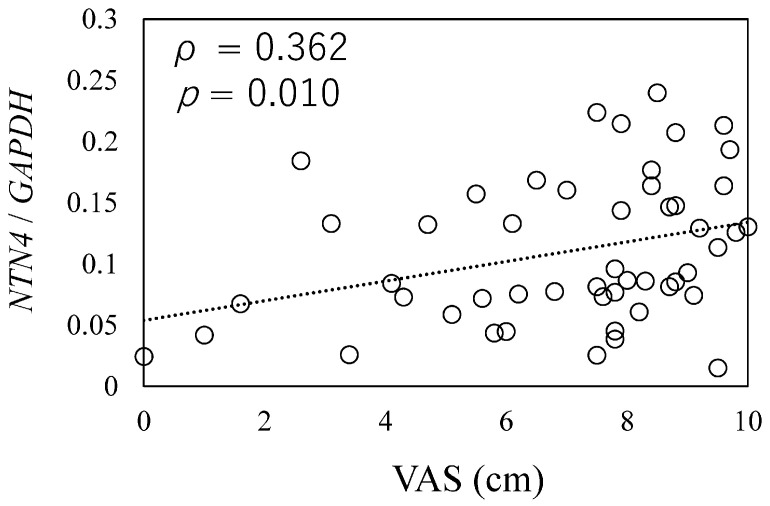
Relationship between *NTN4* expression and osteoarthritis pathology.

To investigate the relationship between *NTN4* expression and pain in OA patients, we analyzed *NTN4* expression levels in synovial tissue samples using qPCR. A positive correlation was observed between *NTN4* expression levels and pain scores, as measured by the VAS (ρ = 0.362, *p* = 0.010, Figure 1).

The correlation between *NTN4* expression and pain score was measured by the Visual Analog Scale (VAS). X-axis: indicates the scores on the Visual Analog Scale (VAS) for pain during movement, as reported by patients. Y-axis: Represents the expression levels of *NTN4* in synovial tissue, normalized to the housekeeping gene *GAPDH*.

Further, a linear regression analysis was conducted to investigate associations between *NTN4* expression and key clinical variables, such as K/L grade, age, BMI, and VAS scores. This analysis revealed a significant association between *NTN4* expression and VAS scores (β = 0.397, *p* = 0.005). In contrast, other variables, including age (β = −0.250, *p* = 0.060), gender (β = −0.250, *p* = 0.079), BMI (β = 0.507, *p* = 0.670), and K/L grade (β = 0.020, *p* = 0.805), did not show significant associations with *NTN4* expression (Table 3).

### 3.2. The Effect of rh-NTN4 on Neurite Outgrowth in Human iPSC-SNs

**Figure 2:**
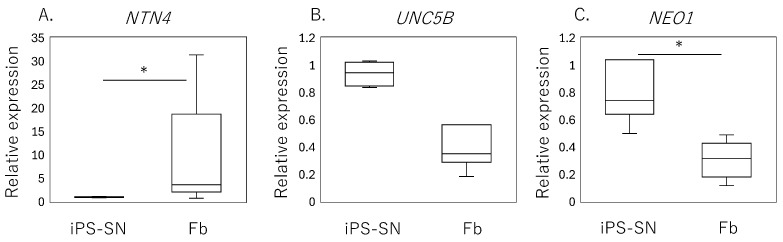
Expression of *NTN4* and its receptors in iPSC-derived sensory neurons (iPSC-SNs) and synovial fibroblasts. Box-and-whisker plots showing the relative expression levels of (**A**) *NTN4*, (**B**) *UNC5B*, and (**C**) *NEO1* in fibroblasts (Fb), normalized to the expression levels in iPSC-derived sensory neurons (iPSC-SNs), which are set as 1. An asterisk (*) indicates statistical significance with *p*-values less than 0.05, demonstrating differences between fibroblast and iPSC-SN expression levels.

qPCR analysis of human iPSC-SNs showed a significantly lower expression of *NTN4* (*p* = 0.010, Figure 2A) compared to the fibroblasts, while no significant differences were observed in the expression levels of *UNC5B* (*p* = 0.065, Figure 2B). However, a higher expression of *NEO1* encoding neogenin was observed in iPSC-SNs (*p* < 0.001, Figure 2C).

**Figure 3:**
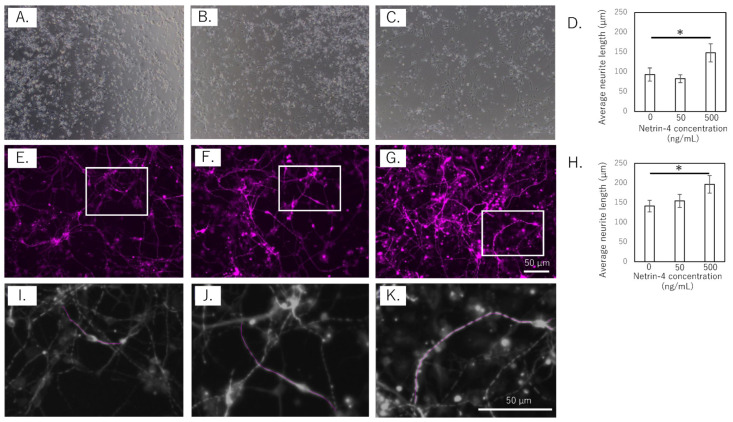
Effect of rh-NTN4 on neurite outgrowth in human iPSC-derived sensory neurons (**A**–**C**). Optical microscopy images at day 4 after rh-NTN4 stimulation: (**A**) vehicle-treated cells, (**B**) cells treated with 50 ng/mL rh-NTN4, (**C**) cells treated with 500 ng/mL rh-NTN4. (**D**) Quantification of neurite length in vehicle-treated, 50 ng/mL rh-NTN4-treated, and 500 ng/mL rh-NTN4-treated cells at day 4. Data are presented as mean ± SE. (**E**–**G**) Fluorescence microscopy images at day 14 after rh-NTN4 stimulation: (**E**) vehicle-treated cells, (**F**) cells treated with 50 ng/mL rh-NTN4, (**G**) cells treated with 500 ng/mL rh-NTN4. (**H**) Quantification of neurite length in vehicle-treated, 50 ng/mL rh-NTN4-treated, and 500 ng/mL rh-NTN4-treated cells at day 14. Data are presented as mean ± SE. * indicates significant differences between groups (*p* < 0.05). (**I**–**K**) Neurite length per neuron in the (**E**–**G**) images was measured using NeuronJ, an ImageJ plug-in.

To assess the effect of rh-NTN4 on neurite outgrowth, the length of the neurites in human iPSC-SNs was measured at 4 and 14 days in vitro (Figure 3A–K). In the short-term culture (4 days), rh-NTN4 treatment significantly enhanced neurite outgrowth compared to the control group (*p* = 0.013, Figure 3A–D). Similarly, in the 14-day culture, sensory neurons treated with rh-NTN4 displayed markedly longer neurites than the untreated controls (*p* = 0.027, Figure 3E–H).

### 3.3. Effect of rh-NTN4 on the Expression of Inflammatory Cytokines, Chemokines, Matrix Metalloproteinases (MMPs), and VCAM1 in Synovial Fibroblasts

**Figure 4:**
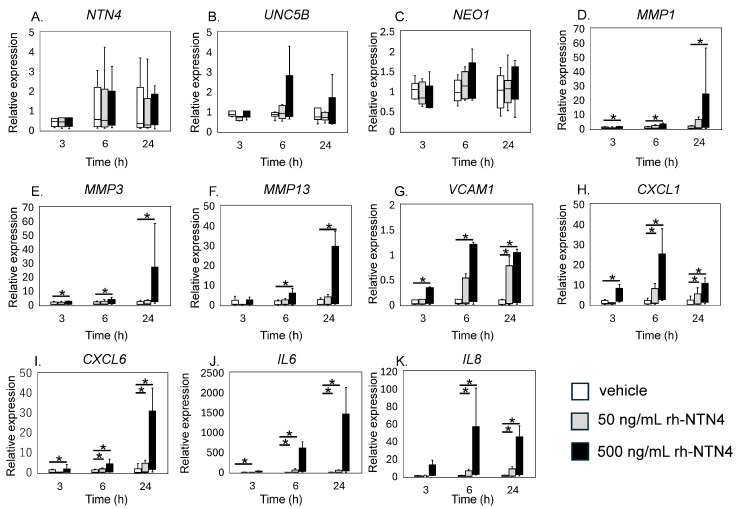
qPCR analysis of vehicle and recombinant Netrin-4 treated fibroblastic cells derived from the synovium. Relative expression levels of (**A**) *NTN4*, (**B**) *UNC5B*, (**C**) *NEO1*, (**D**) *MMP1*, (**E**) *MMP3*, (**F**) *MMP13*, (**G**) *VCAM1*, (**H**) *CXCL1*, (**I**) *CXCL6*, (**J**) *IL6*, and (**K**) *IL8* following rh-NTN4 treatment compared to vehicle control is presented using box-and-whisker plots. These plots depict the median, quartiles, and range of each dataset. Statistical significances between groups are clearly indicated with lines, and asterisks (*) denote *p*-values less than 0.05, highlighting statistically significant differences.

qPCR analysis revealed that exogenous rh-NTN4 stimulation did not alter the expression of endogenous *NTN4*, *UNC5B*, or *NEO1* (Figure 4A–C). Following the initial analysis, we evaluated the effects of rh-NTN4 on synovial fibroblasts, drawing on insights from our previous RNA-Seq analysis of infrapatellar fat pad-derived stromal cells treated with rh-NTN4 [23]. *MMP1* and *MMP3* exhibited significant differences in expression between the 0 ng/mL and 500 ng/mL groups at all time points (*MMP1*: 3 h, *p* = 0.001; 6 h, *p* < 0.001; 24 h, *p* < 0.001; *MMP3*: 3 h, *p* = 0.011; 6 h, *p* = 0.001; 24 h, *p* < 0.001) (Figure 4D,E). Similarly, *MMP13* showed significant differences at 6 h (*p* < 0.001) and 24 h (*p* = 0.003) (Figure 4F). *VCAM1* expression was significantly different between the 0 ng/mL and 500 ng/mL groups at 3 h (*p* = 0.016), 6 h (*p* = 0.001), and 24 h (*p* < 0.001), and between the 0 ng/mL and 50 ng/mL groups at 24 h (*p* = 0.046) (Figure 4G).

*CXCL1* and *CXCL6* both displayed significant differences between the 0 ng/mL and 500 ng/mL groups at 3, 6, and 24 h (*CXCL1*: all *p* < 0.001; *CXCL6*: 3 h, *p* = 0.024; 6 h, *p* < 0.001; 24 h, *p* < 0.001), with additional differences observed between the 0 ng/mL and 50 ng/mL groups at 6 h and 24 h (*p* = 0.046 for both markers) (Figure 4H,I). The *IL6* and *IL8* expression levels were also significantly different between the 0 ng/mL and 500 ng/mL groups at all time points (*p* < 0.001), and between the 0 ng/mL and 50 ng/mL groups at 6 h and 24 h (*p* = 0.046) (Figure 4J,K).

**Figure 5:**
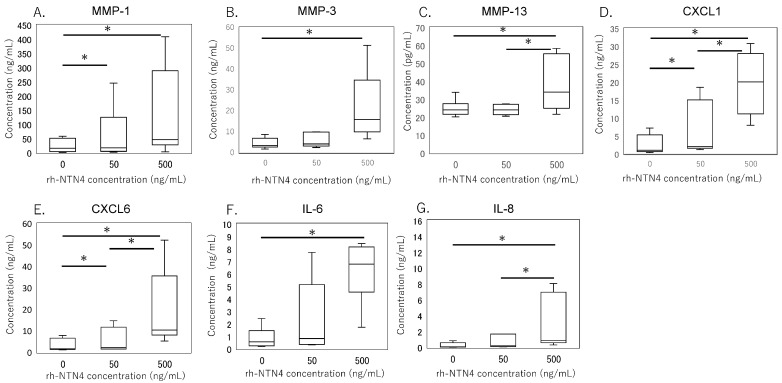
ELISA analysis of cell supernatant in vehicle- and recombinant Netrin-4-treated fibroblastic cells derived from the synovium. Concentrations of (**A**) MMP-1, (**B**) MMP-3, (**C**) MMP-13, (**D**) CXCL1, (**E**) CXCL6, (**F**) IL-6, and (**G**) IL-8 in cell supernatant are presented in box-and-whisker plots, showing the median, 25th and 75th percentiles, and range. * *p* < 0.05.

**Figure 6:**
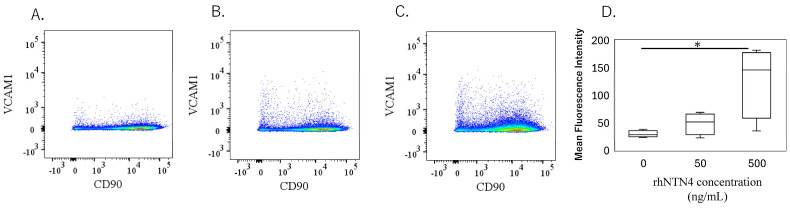
Flow cytometric analysis of vehicle- and recombinant Netrin-4-treated fibroblastic cells derived from the synovium. (**A**–**C**): Dot plot analysis of fibroblastic cells treated with vehicle (**A**), 50 ng/mL (**B**), and 500 ng/mL (**C**) recombinant human Netrin-4 (rh-NTN4). (**D**) Mean fluorescence intensity of vehicle- and rhNTN4-treated fibroblastic cells displayed as box-and-whisker plots. * indicates significant differences (*p* < 0.05) vs. vehicle.

To validate the qPCR data, ELISA was performed on culture supernatants. MMP-1 levels were significantly higher with 50 ng/mL rh-NTN4 treatment (*p* = 0.024) and further increased at 500 ng/mL rh-NTN4 (*p* < 0.001) (Figure 5A). MMP-3 levels demonstrated a significant rise at 500 ng/mL rh-NTN4 (*p* < 0.001) (Figure 5B). Similarly, MMP-13 levels were significantly elevated at 500 ng/mL compared to the vehicle control (*p* = 0.037) (Figure 5C). Furthermore, CXCL1 levels increased significantly with 50 ng/mL rh-NTN4 (*p* = 0.046) and were further elevated at 500 ng/mL (*p* < 0.001) (Figure 5D). Likewise, CXCL6 demonstrated a significant elevation at both 50 ng/mL (*p* = 0.046) and 500 ng/mL rh-NTN4 (*p* < 0.001) (Figure 5E). Both IL-6 and IL-8 levels were also significantly elevated in the presence of 50 ng/mL rh-NTN4 (IL-8: *p* = 0.046) and 500 ng/mL rh-NTN4 (IL-6: *p* < 0.001; IL-8: *p* < 0.001) compared to vehicle control (Figure 5F,G). To validate the qPCR result of VCAM1 expression, flow cytometric analysis was also performed (Figure 6A–D). The MFI in 500 ng/mL rh-NTN4 was significantly higher than that in vehicle control (*p* = 0.040) (Figure 6D).

## 4. Discussion

This study advances our understanding of the role of NTN4 in KOA by exploring its potential links to pain severity and its effects on synovial fibroblasts and human iPS-SNs. Our results demonstrate a significant correlation between NTN4 levels in the synovium and pain scores. Furthermore, NTN4 was found to promote axonal growth in iPS-SNs and to stimulate the production of inflammatory mediators in synovial fibroblasts. These findings suggest that NTN4 plays a complex role in KOA, potentially influencing both neuronal sensitization and inflammatory responses. Further investigation into NTN4-mediated neuronal and inflammatory pathways could provide deeper insights into how NTN4 contributes to OA pain mechanisms.

Previous research has shown that synovium, a richly innervated tissue, plays a pivotal role in pain perception in KOA [13,31,32] NTN4’s influence on sensory neurons, particularly in promoting neurite outgrowth, underscores its potential role in sensitization(ref. This study also demonstrated that NTN4 can affect neuronal growth and branching via the UNC5B receptor in rats, impacting pain sensitivity [18]. While Netrin-4’s impact on sensory neurons was previously evaluated using mouse DRG neurons [17], its effects on human cells have not been clarified. In this study, we utilized human iPSC-SNs to further explore NTN4’s biological roles. Our findings confirm the expression of NTN4 receptors, *NEO1* and *UNC5B*, in human-derived neurons, and we observed enhanced axonal elongation similar to that seen in mouse cells [17]. These observations suggest that NTN4 could play a similar role in promoting sensory nerve outgrowth within the human synovium, potentially increasing the density of pain-transmitting fibers. However, it is important to note that while these results are promising, they primarily provide a foundation for hypothesizing about NTN4’s function in human OA. The actual impact on pain perception in KOA patients remains to be directly demonstrated. The hypothesis that NTN4 may amplify pain signals by increasing sensory nerve innervation aligns with broader research linking increased sensory innervation to heightened pain sensitivity in OA-affected joints [33]. Future studies are necessary to elucidate the precise mechanisms by which NTN4 may influence pain pathways in human OA.

Previous studies have shown that the severity of synovitis correlates with increased knee pain, highlighting a connection between inflammation and nociceptive activity [34,35,36,37,38]. IL-6 plays a critical role in the development and persistence of hyperalgesia across various pain models [39]. Elevated IL-6 levels in the spinal cord have been shown to induce mechanical hyperalgesia in rats and are linked to nociceptive sensory processes [40,41]. Dysregulation of IL-6 leads to the production and release of various inflammatory mediators, which can activate neurocytes and potentially contribute to neuropathic pain [24,39]. Targeting IL-6 signaling has been shown to result in significant clinical improvements in inflammatory arthritis [42]. Similarly, elevated plasma IL-8 concentrations, which reflect higher levels of peripheral inflammation, have been associated with lower pressure pain thresholds in OA patients [43]. Our findings indicate that NTN4 could potentially play a role in these processes by promoting axonal growth in sensory neurons and upregulating pro-inflammatory cytokines and chemokines in synovial fibroblasts. However, while these results are promising, they should be interpreted with caution. The direct causal relationships between NTN4 action, inflammatory mediator production, and pain perception in KOA have not been definitively established. This study suggests that NTN4 might contribute to local inflammation and neuronal sensitization, potentially exacerbating pain perception in KOA, but further research is necessary to confirm these preliminary findings and understand the underlying mechanisms.

In our study, NTN4 was identified as playing a role in promoting the expression of MMP1, MMP3, and MMP13, all of which are involved in extracellular matrix degradation [44]. These MMPs play a pivotal role in the breakdown of collagen and proteoglycans, leading to the progressive deterioration of synovial tissue integrity in OA. By driving the expression of these catabolic enzymes, NTN4 may contribute to the pathological remodeling of the synovial microenvironment observed in OA. Targeting NTN4 could, therefore, offer a novel therapeutic strategy to mitigate matrix degradation and preserve synovial and joint health. This lack of variation may be attributed to this study’s focus on end-stage OA, where synovial inflammation and ECM degradation are already at advanced levels, potentially obscuring differences that might be more evident in earlier stages of disease progression. The role of NTN4 in the earlier phases of OA, where ECM turnover is initiated and inflammatory responses are developing, remains unexplored and could provide critical insights into its involvement in the initiation and progression of OA pathology. Examining NTN4 activity in normal or early-stage OA synovium could clarify whether its upregulation serves as an early trigger for pathological processes, such as the recruitment of inflammatory cells, nociceptor sensitization, and ECM breakdown. Furthermore, early-stage studies might reveal whether NTN4 inhibition has the potential to slow or even prevent OA progression by preserving synovial integrity and dampening inflammatory responses before irreversible joint damage occurs.

Several limitations warrant further attention in future studies. First, our study did not assess whether iPSC-SNs express nociceptive neuropeptides such as Substance P, nor did we explore how NTN4 stimulation might alter such expression. This is crucial for fully understanding NTN4’s role in neuronal sensitization and pain mechanisms in OA. Second, this study was limited in exploring the interactions between NTN4 and other inflammatory mediators, which could provide deeper insights into its regulatory mechanisms. Additionally, examining NTN4’s effects on fibroblast behavior, matrix degradation, and crosstalk with cartilage cells was not conducted, which would offer a more comprehensive understanding of its role in OA pathology.

Finally, further research involving larger, diverse patient cohorts and healthy controls is essential to enhance our understanding of NTN4’s systemic effects on joint tissues and its specific role in modulating pain mechanisms in OA. This should include studies using a variety of tissue samples, such as those obtained from synovial fluid punctures, and utilizing a broad range of patient samples along with the Osteoarthritis Research Society International (OARSI) staging system [45] to elucidate the complex dynamics of NTN4 in relation to the inflammatory milieu and pain pathways at different stages of OA. This could elucidate the complex dynamics of NTN4 and its relationship with the inflammatory milieu and pain pathways at different stages of OA.

## 5. Conclusions

This study suggests a potential correlation between NTN4 levels in the synovium and pain severity in KOA, indicating a role for NTN4 in pain modulation. Our findings show that NTN4 promotes axonal growth in human iPSC-derived sensory neurons and stimulates the release of pro-inflammatory cytokines in synovial fibroblasts. These results may imply that NTN4 could exacerbate pain by enhancing neuronal growth and inflammatory mediator release. Further research is essential to fully explore how NTN4-mediated effects contribute to the mechanisms of OA pain.

## Figures and Tables

**Table 1:** Primer sequences.

Gene		Sequence	bp
*CXCL1*	sense	GCT TGC CTC AAT CCT GCA TC	73
	antisense	AGT TGG ATT TGT CAC TGT TCA GC	
*CXCL6*	sense	GGT CCT TCG GGC TCC TTG TG	125
	antisense	ACG CGT AAA CAA GTG CAA CG	
*IL6*	sense	GAG GAG ACT TGC CTG GTG AAA	199
	antisense	TGG CAT TTG TGG TTG GGT CA	
*IL8*	sense	ACA CTG CGC CAA CAC AGA AA	89
	antisense	CAA CCC TCT GCA CCC AGT TT	
*MMP1*	sense	ACT TAC ATC GTG TTG CGG CT	164
	antisense	CGA TGG GCT GGA CAG GAT TT	
*MMP3*	sense	GTG GAG TTC CTG ACG TTG GT	164
	antisense	TGG AGT CAC CTC TTC CCA GA	
*MMP13*	sense	TGA CTG AGA GGC TCC GAG AA	111
	antisense	CAT CAG GAA CCC CGC ATC TT	
*NEO1*	sense	GGGCATGAGTCAGAGGACAG	127
	antisense	CGAGGGAATGGATGGGATGG	
*NTN4*	sense	TGT TGT CAA GAA GGG CGC TA	159
	antisense	ACG CGA AGG TTG GTG ATCT T	
*UNC5B*	sense	CAGAACGACCACGTCACACA	121
	antisense	ACCAGTAATCCTCCAGCCCA	
*VCAM1*	sense	CCA TCC ACA AAG CTG CAA GA	70
	antisense	CTG GAG CTG GTA GAC CCT CG	

**Table 2:** Clinical characteristics of knee osteoarthritis patients.

	All (*n* = 50)	Male (*n* = 13)	Female (*n* = 37)	*p*-Value
Age (years)	75.0 ± 7.5	71.9 ± 6.4	75.8 ± 7.6	0.174
BMI (kg/m^2^)	26.6 ± 4.1	26.6 ± 3.2	26.6 ± 4.4	0.805
KL grade (2/3/4), n	2/8/40	0/0/13	2/8/4	0.648
VAS (cm)	7.0 ± 2.4	7.5± 1.8	6.9± 2.6	0.311

BMI, body mass index; KL grade, Kellgren–Lawrence grade; VAS., Visual Analog Scale.

**Table 3:** Linear regression analysis of variables associated with *NTN4* expression.

Variable	β	*p*-Value
Age (years)	−0.266	0.060
Gender, male/female	−0.250	0.079
BMI (kg/m^2^)	0.089	0.507
KL grade	0.020	0.884
VAS (mm)	0.397	0.005

## Data Availability

The original contributions presented in this study are included in the article. Further inquiries can be directed to the corresponding author(s).
